# The Outcome of Endoscopic Transethmosphenoid Optic Canal Decompression for Indirect Traumatic Optic Neuropathy with No-Light-Perception

**DOI:** 10.1155/2016/6492858

**Published:** 2016-11-14

**Authors:** Bo Yu, Yingjie Ma, Yunhai Tu, Wencan Wu

**Affiliations:** ^1^Department of Orbital & Oculoplastic Surgery, Eye Hospital of Wenzhou Medical University, Wenzhou, Zhejiang, China; ^2^Eye Hospital of Wenzhou Medical University, Wenzhou, Zhejiang, China

## Abstract

*Purpose*. To present the safety and effect of endoscopic transethmosphenoid optic canal decompression (ETOCD) for indirect traumatic optic neuropathy (ITON) patients with no-light-perception (NLP).* Methods*. A retrospective study performed on 96 patients (96 eyes) with NLP after ITON between June 1, 2010, and June 1, 2015, who underwent ETOCD, was reviewed. Visual outcome before and after treatment was taken into comparison.* Results*. The overall visual acuity improvement rate after surgery was 46.9%. The improvement rates of visual acuity of patients who received treatment within 3 days of injury, 3–7 days after injury, and later than 7 days were 63.6%, 42.9%, and 35.7%, respectively. Statistically significant difference was detected between the effective rates of within-3-day group and later-than-7-day group (*χ* ^2^ = 5.772, *P* = 0.016). The effective rate of atrophy group and nonatrophy group was 25.0% and 51.3%, respectively. The effective rate was significantly higher in nonatrophy group (*χ* ^2^ = 4.417, *P* = 0.036).* Conclusion*. For patients suffering from ITON with NLP, time to medical treatment within 3 days is an influential factor for visual prognosis. Optic nerve atrophy is an important predictor for visual prognosis. Treatment should still be recommended even for cases of delayed presentation to hospital.

## 1. Introduction

Traumatic optic neuropathy (TON) is a rare but serious complication after head trauma. It occurs in about 2.5% of midface fractures and 10% of craniofacial fractures [1–4]. The initial clinical findings of TON include decrease of vision acuity, a relative afferent pupillary defect. Optic nerve atrophy always occurs weeks or even months after injury.

TON can be caused by direct or indirect injury. Direct optic injury usually results from optic nerve avulsion or laceration, or from direct fracture of the optic canal. Indirect optic injury is caused by increased intracanalicular pressure after an injury, which usually initiates a cascade of molecular and chemical mediators leading to secondary disorders [5]. The prognosis of direct optic injury is usually quite poor. But indirect injuries, such as edema, hematoma, or moderate bony optic nerve compression, may derive benefit from treatment [6].

Visual acuity of ITON ranges from normal to no-light-perception. Approximately 50% have permanent vision loss [4, 7, 8]. About 40% to 60% of ITON patients have severe visual loss of light perception or worse at baseline [4, 9–12]. The initial visual acuity is a strong predictor of prognosis. Visual recovery was thought to be particularly poor when the initial vision was NLP [10].

The management for ITON is still controversial. ETOCD was a new treatment for it which raised up this decade by some ENTs.

In this case series, we set out to explore the safety and effect of ETOCD for ITON patients with NLP.

## 2. Patients and Methods

The study was authorized by the Eye Hospital of Wenzhou Medical University and followed the principles outlined in the Declaration of Helsinki (2008). It was approved by the Institutional Ethics Committee (KYK[2016]18) (Medical Ethics Committee, Wenzhou Medical University, Wenzhou, Zhejiang, China).

Medical records of all NLP patients after ITON without brain trauma, aged 6–62 years old, who were treated in Department of Orbital & Oculoplastic Surgery, Eye Hospital of Wenzhou Medical University between June 1, 2010, and June 1, 2015, were reviewed. Patients with bilateral ITON were not included because these patients may be accompanied by cranial cerebral injury or optic chiasm injury. Patients who had previous treatment were also excluded in our study.

The diagnosis of ITON was made by the presence of vision loss after direct or indirect head trauma, with a relative afferent pupillary defect, and could not be explained by other causes. If necessary, visual evoked potential (VEP) was performed for diagnosis. High-resolution computed tomography (HRCT) scan of the head and the orbit was performed in all the patients. OCT scan and fundus photo were taken to evaluate the optic nerve as well. All cases after admission were administered by methylprednisolone (20 mg/kg/day) and mouse-derived nerve growth factor (NGF) (30 *μ*g/mL/day) (Staidson Beijing) for 3 days. And then patients whose VA showed no improvement after intravenous treatment were recommended to ETOCD. Patients were followed up to 3 months after surgery.

We recorded the age of the patients, VA before surgery, VA at the 3 months after surgery, time to medical treatment, and time of visual loss, together with other complications like consciousness impairment, hemorrhage within the postethmoid and/or sphenoid sinus, orbital fracture, and optic canal fracture. A patient's VA was considered to have improved if an improvement was from no-light-perception to light perception or better.

A total 147 patients meet our criterion at first, but then 51 patients were excluded. 26 of them gained VA improvement after 3 days of methylprednisolone and NGF and was not subjected to ETOCD. Another 6 patients with no VA improvement refused the surgery. Four patients who had incomplete preoperative exam were excluded from all analyses. Follow-up data were incomplete for 15 patients who were excluded from all analyses as well. The rest 96 surgery patients were divided into three groups (within 3 days, 3–7 days, and later than 7 days of injury) according to the interval between the first treatment and injury. Enrolled surgery cases were divided into atrophy group and nonatrophy group according to optic nerve situation (evaluated by OCT and fundus photo) before surgery as well.

### 2.1. Surgical Procedure

**Figure 1:**
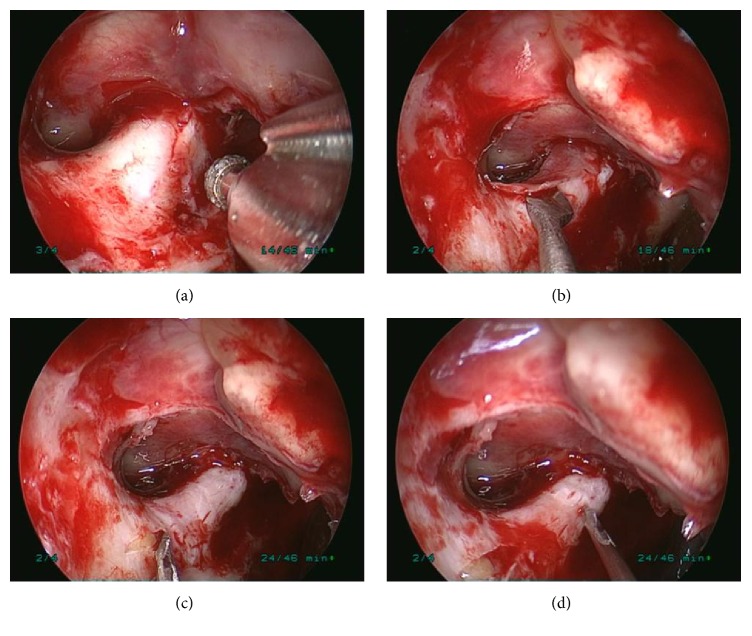
The surgical procedures of ETOCD. (a) The medical wall of optic canal was thinned by a microdrill. (b) The thinned medial wall of optic canal was removed by a microcurette. (c) The periorbita of the orbital apex and annulus of Zinn was split with a sharp 9# MVR knife. (d) Five to six small intermittent punctuated incisions were made to the optic nerve sheath.

All of the procedures were performed under general anesthesia by a single orbital surgeon (Dr. Wencan Wu). During endoscopic optic nerve decompression, a routine endoscopic sphenoethmoidectomy was performed using the Messerklinger technique with preservation of the middle turbinate. The sphenoid face is opened widely, and the bulge of the optic nerve canal is identified along the lateral wall of the sphenoid sinus, superior to the carotid artery. In some patients, the optic canal may be identified initially in a posterior ethmoid or Onodi cell, which can be identified on preoperative CT scan. Identification and opening of the Onodi cell are important to provide adequate surgical exposure and allow full access to the optic canal. In some children, the sphenoid sinus was not fully developed; in order to locate the optic nerve canal, the navigation system was used during surgeries. After complete sphenoethmoidectomy, the lesser wing of the sphenoidal bone and the medial wall of the optic canal, which spans from the orbital aperture to the cranial cavity, were thinned with a microdrill and removed with a microcurette. Then, the periorbita of the orbital apex, annulus of Zinn, and the optic nerve sheath were incised with a sharp 9# MVR knife. Finally, the operating field of the optic canal was covered by a piece of sterile gelatin sponge that was immersed in dexamethasone (5 mg/2 mL) and mouse-derived NGF (30 *μ*g/mL). Postoperative care included administering methylprednisolone (20 mg/kg/day) for 4 days, ceftriaxone (2.0 g/day) for 5 days, and NGF (30 *μ*g/mL/day) for 1 month (Figure 1).

### 2.2. Statistical Analysis

Statistical analyses were performed with SPSS version 17.0. Effective rate was defined as the number of patients with improved VA after ETOCD divided by overall number of patients enrolled in our study. *χ* ^2^ test was used to compare effective rate between within-3-day group, 3–7-day group, and later-than-7-day group and atrophy and nonatrophy group. Results were considered significant at *P* < 0.05.

## 3. Results

Among the 96 children (96 eyes), 86 were male and 10 were female. The age ranged from 6 to 62 years with a mean age of 31.2 ± 14.7 years. The most common cause of ITON in our study was fall (48/96), followed by car accident (36/96) and assaults (11/96). 48 patients showed consciousness impairment after the trauma. Orbital bone fracture was found in 33 patients and hemorrhage within the postethmoid and/or sphenoid sinus was found in 39 patients. There were 37 cases with optic canal fracture shown on image scan and another 21 detected during the surgery. 33 patients were admitted to our hospital for treatment within 3 days after trauma. In comparison, 21 patients received treatment between 3 and 7 days after trauma, while another 42 patients received treatment later than 7 days (Table 1).

Visual acuity was improved in 45 out of 96 patients who underwent ETOCD, with a total effective rate of 46.9%.

The effective rate of within-3-day group was 63.6%. The effective rate of 3–7-day group was 42.9%. The effective rate of later-than-7-day group was 35.7%. Statistically significant difference was detected between the effective rates between within-3-day group and later-than-7-day group (*χ* ^2^ = 5.772, *P* = 0.016). No statistical difference was detected between the effective rates between within-3-day group and 3–7-day group and 3–7-day group and later-than-7-day group. (*χ* ^2^ = 2.244, *P* = 0.134; *χ* ^2^ = 0.303, *P* = 0.582, resp.).

The effective rate of atrophy group was 25.0%. The effective rate of nonatrophy group was 51.3%. The effective rate was significant higher in nonatrophy group (*χ* ^2^ = 4.417, *P* = 0.036).

During the surgery, 4 patients developed cerebrospinal fluid rhinorrhea (CSFR). Three of them were repaired by mucosal flap transplantation uneventfully during surgery and one resolved from strict bed rest in 30 degrees of head-up and feet-down position. One experienced cavernous sinus hemorrhage during the surgery, which was controlled by pressing and packing hemostasis. No other severe complications were observed.

## 4. Discussion

Indirect traumatic optic neuropathy (ITON) is a rare but serious complication after head trauma. Motor vehicle is the most common cause of ITON, followed by bicycle accident, falls, and assaults [9, 10]. The most common cause of ITON in our study was falls (48/96), followed by car accident (36/96), assaults (11/96), and others (1/96).

Treatment for ITON remains controversial and no definite conclusion has been reached yet. According to previous studies, the VA improvement rate of high-dose steroid treatment and high-dose steroid combined with OCD was 4.3–44% and 60.9–71.1% [13–17]. OCD with combination of steroid is generally thought to be more effective in visual improvement than steroid treatment only. OCD physically decompresses the nerve within the canal, thereby creating space for the nerve to swell and limiting the damaging effect of the compartment syndrome [13]. The endoscopic approach offers many advantages, including decreased morbidity, preservation of olfaction, ideal cosmetic results without external scarring, no risk of injury to the developing teeth in children, and a shorter recovery time. Most importantly, the endoscopic approach provides an excellent view of the orbital apex [14].

Whether to split the nerve sheath during ETOCD is another controversial issue. Thakar A. believed that the annulus of Zinn at the anterior end of the fibrous sheath may contribute to edema [18]. However, slitting of the sheath may increase the risk of cerebrospinal fluid leakage, incidence of ophthalmic artery injury, and secondary injury to the optic nerve. In our study, all patients in surgery group underwent optic nerve sheath splitting during the surgery. We performed punctuated optic nerve sheath splitting in this study. This procedure is different from the traditional way of slitting the optic nerve sheath, which is associated with complications like cerebrospinal fluid leakage, ophthalmic artery injury, and optic nerve injury. In our procedure, 5 to 6 small intermittently punctuated incisions were made to the optic nerve sheath to further release the pressure with little risk of complications. Meanwhile, the splitting can create extra space for local administration of dexamethasone and mouse-derived NGF. The medicine would accumulate in sphenoid sinus for slow releasing and promote optic nerve rehabilitation continually. We also decompress the orbit apex by opening the periorbital of the orbital apex and annulus of Zinn to further decrease intracanal pressure.

The baseline visual acuity is predictive for VA improvement after surgery. An improvement of 60.9–71.1% after ETOCD has been reported [13, 14, 17]. When it comes to patients with NLP, the effective rate dropped from 10% to 30% [19–21]. Herein we reported an overall effective rate of 46.9% after ETOCD in ITON patients with NLP before surgery.

Some report that patients receiving early treatment (equal or less than 7 days) will have better VA prognosis [22, 23]. We found the effective rate of early treat (less than 3 days) was 63.6% which was higher than late treated cases (later than 7 days, 35.7%). Statistical significance was detected between early treated cases and delayed treated cases. 35.7% of late treated patients had improved VA after ETOCD, which reminds us that we should never give up ITON patients with NLP too early even when they are delayed for treatment.

Optic nerve atrophy is another important predictor for ITON. We found 51.3% of effective rate in patients with nonatrophic optic nerve which is much higher than those with atrophic optic nerve (25.0%). It indicated that the nonatrophic optic nerve is crucial for VA prognosis and patients with nonatrophic optic nerve may benefit more from the surgical intervention, since atrophic optic nerve implies the probable degeneration and apoptosis of ganglionic cells of the retina [24].

## 5. Conclusion

For patients suffering from ITON with NLP, time to medical treatment within 3 days is an influential factor for visual prognosis. Optic nerve atrophy is an important predictor for visual prognosis. No severe complication is encountered. Large-sampled controlled prospective researches are needed to further prove the rationality of ETOCD for ITON patient with NLP and to work out the exact surgical indication.

## Figures and Tables

**Table 1:** Clinical characteristic of 96 NLP patients with ITON.

Characteristic	Case (*n*) (%)
Injury type	
Fall	48 (50.0)
Car accident	36 (37.5)
Assault	11 (11.5)
Explosion	1 (1.0)
State of consciousness	
Impairment	48 (50.0)
None	48 (50.0)
Orbital bone fracture	
Yes	33 (34.4)
No	63 (65.6)
Hemorrhage within the postethmoid and/or sphenoid sinus	
Yes	39 (40.6)
No	57 (59.4)
Optic canal fracture	
On image scan	37 (38.5)
Found during surgery	58 (60.4)
None	38 (39.6)
Time to medical treatment	
Within 3 days	33 (34.4)
3–7 days	21 (21.9)
After 7 days	42 (43.8)
Optic nerve	
Atrophic optic nerve	20 (20.8)
Nonatrophic optic nerve	76 (79.2)
